# Supplementary material for: Sequence variation and linkage disequilibrium in the GABA transporter-1 gene (SLC6A1) in five populations: implications for pharmacogenetic research
Source: BMC Genet. 2007 Oct 17;8:71. doi: 10.1186/1471-2156-8-71 (PMC2175509; doi:10.1186/1471-2156-8-71)
Supplement: Additional file 1 — List of SNPs discovered in SLC6A1 by resequencing 40 individuals from Thai (n = 8), Hmong (n = 8), European-American (n = 7), African-American (n = 9) and Finnish (n = 8) populations. The data provided descriptions of all SNPs in SLC6A1 in the population samples. [file 1471-2156-8-71-S1.doc]

**Additional data file**

List of SNPs discovered in *SLC6A1* by resequencing 40 individuals from Thai (n=8), Hmong (n=8), European-American (n=7), African-American (n=9) and Finnish (n=8) populations.

| **Name** | **rs** | **position** | **location** | **alleles** | **flanking sequences** | **EA** | **AA** | **Finn** | **Thai** | **Hmong** |
| --- | --- | --- | --- | --- | --- | --- | --- | --- | --- | --- |
| -29477 | rs1710879 | 11004420 | 5’upstream | T/C | TCATA**T/C**GTAGTT | 0.71/0.29 (0.197-0.383) | 0.99/0.01  (-0.008-0.028) | 0.75/0.25 (0.162-0.338) | 0.76/0.24 (0.163-0.317) | 0.78/0.22 (0.137-0.303) |
|  |  |  |  |  |  |  |  |  |  |  |
| -25070 | rs2675156 | 11008827 | 5’upstream | A/C | ACCCC**A/C**ACCCA | 0.64/0.36 (0.109-0.611) | 0.28/0.72 (0.073-0.487) | 0.63/0.37  (0.133-0.607) | 0.63/0.37 (0.133-0.607) | 0.75/0.25 (0.038-0.462) |
|  |  |  |  |  |  |  |  |  |  |  |
| -24993 | rs2697151 | 11008904 | 5’upstream | A/G | GCAGT**A/G**CTGCA | 0.36/0.64 (0.109-0.611) | 0/1 | 0.19/0.81  (-0.002-0.382) | 0.13/0.87  (-0.035-0.295) | 0/1 |
|  |  |  |  |  |  |  |  |  |  |  |
| -24924 | rs2675157 | 11008973 | 5’upstream | A/G | GACCA**A/G**GACGG | 0.64/0.36 (0.109-0.611) | 0.61/0.39 (0.165-0.615) | 0.63/0.37  (0.133-0.607) | 0.94/0.06  (-0.056-0.176) | 0.81/0.19  (-0.002-0.382) |
|  |  |  |  |  |  |  |  |  |  |  |
| -24794 | rs41469948 | 11009103 | 5’upstream | A/G | AGGGA**G/A**GGAGG | 0/1 | 0.39/0.61 (0.165-0.615) | 0/1 | 0/1 | 0/1 |
|  |  |  |  |  |  |  |  |  |  |  |
| -24788 | rs41342644 | 11009109 | 5’upstream | ins21bp | **[GGGTGGGGGAGAG** | 0/1 | 0.39/0.61  (0.165-0.615) | 0/1 | 0/1 | 0/1 |
|  |  |  |  |  | **GGAGGGAGG]** |  |  |  |  |  |
| -24780 | rs41539860 | 11009117 | 5’upstream | GG/-GG | AGAGG**[GG]**AGAAA | 0.70/0.30  (0.06-0.54) | 0.77/0.23 **^b^** (0.036-0.424) | 0.67/0.33 (0.100-0.560) | 0.78/0.22 (0.017-0.423) | 0.84/0.16  (-0.02-0.34) |
|  |  |  |  |  |  |  |  |  |  |  |
| -24534 | rs41338544 | 11009363 | 5’upstream | C/T | GCGGG**C/T**CCTGC | 1/0 | 1/0 | 0.94/0.06  (-0.056-0.176) | 1/0 | 1/0 |
|  |  |  |  |  |  |  |  |  |  |  |
| -24343 | rs41429044 | 11009554 | Exon1 (5’UTR) | C/T | GCGCA**T/C**CGGAG | 0/1 | 0/1 | 0.06/0.94  (-0.056-0.176) | 0/1 | 0/1 |
|  |  |  |  |  |  |  |  |  |  |  |
| -24321 | rs34189945 | 11009576 | Exon1 (5’UTR) | A/C | CCCGG**A/C**GCAGC | 0.64/0.36 (0.109-0.611) | 0.33/0.67 (0.113-0.547) | 0.75/0.25 (0.038-0.462) | 0.69/0.31 (0.083-0.537) | 0.75/0.25 (0.038-0.462) |
|  |  |  |  |  |  |  |  |  |  |  |
| -24321 | rs34189945 | 11009576 | Exon1 (5’UTR) | A/C | CCCGG**A/C**GCAGC | 0.66/0.34 (0.243-0.437) | 0.59/0.41 (0.322-0.498) | 0.68/0.32 (0.226-0.414) | 0.64/0.36 (0.273-0.447) | 0.66/0.34 (0.245-0.435) |
|  |  |  |  |  |  |  |  |  |  |  |
| -24271 | rs41486951 | 11009627 | Intron1 | A/G | AGGGC**G/A**GCCGG | 0/1 | 0/1 | 0/1 | 0.06/0.94  (-0.056-0.176) | 0/1 |
|  |  |  |  |  |  |  |  |  |  |  |
| -24126 | rs41506145 | 11009771 | Intron1 | G/T | CCACA**T/G**GAAGG | 0/1 | 0.33/0.67 (0.113-0.547) | 0/1 | 0/1 | 0/1 |
|  |  |  |  |  |  |  |  |  |  |  |
| -17590 | rs1568074 | 11016307 | Intron1 | T/C | GAGGC**T/C**TGGGC | 0.51/0.49 (0.388-0.592) | 0.22/0.78 (0.146-0.294) | 0.59/0.41 (0.311-0.509) | 0.5/0.5  (0.410-0.590) | 0.51/0.49 (0.390-0.590) |
|  |  |  |  |  |  |  |  |  |  |  |
| -13071 | rs1710892 | 11020826 | Intron1 | G/A | TCTGC**G/A**GTTTT | 0.53/0.47 (0.368-0.572) | 0.31/0.69 (0.227-0.393) | 0.63/0.37  (0.272-0.468) | 0.5/0.5  (0.410-0.590) | 0.5/0.5  (0.400-0.600) |
|  |  |  |  |  |  |  |  |  |  |  |
| -9765 | rs1710891 | 11024132 | Intron1 | C/T | CAGGA**C/T**TGGAT | 0.55/0.45 (0.348-0.552) | 0.5/0.5  (0.411-0.589) | 0.6/0.4  (0.301-0.499) | 0.53/0.47 (0.380-0.560) | 0.63/0.37 (0.237-0.467) |
|  |  |  |  |  |  |  |  |  |  |  |
| -1569 | rs41422847 | 11032328 | Intron1 | C/T | CATCC**C/T**TCCTC | 1/0 | 1/0 | 1/0 | 1/0 | 0.94/0.06  (-0.056-0.176) |
|  |  |  |  |  |  |  |  |  |  |  |
| -1529 | rs6342 | 11032368 | Exon2 (5’UTR) | A/G | ACCCA**G/A**GGTGG | 0.43/0.57 (0.171-0.689) | 0.11/0.89  (-0.035-0.255) | 0.44/0.56 (0.197-0.683) | 0.44/0.56 (0.197-0.683) | 0.06/0.94  (-0.056-0.176) |
|  |  |  |  |  |  |  |  |  |  |  |
| -1529 | rs6342 | 11032368 | Exon2 (5’UTR) | A/G | ACCCA**G/A**GGTGG | 0.48/0.52 (0.378-0.582) | 0.22/0.78 (0.146-0.294) | 0.67/0.33 (0.235-0.425) | 0.34/0.66 (0.255-0.425) | 0.14/0.86  (0.071-0.209) |
|  |  |  |  |  |  |  |  |  |  |  |
| -1213 | rs41386254 | 11032684 | Intron2 | A/G | GCCCA**A/G**TTTCC | 1/0 | 0.94/0.06  (-0.05-0.17) | 1/0 | 1/0 | 1/0 |
|  |  |  |  |  |  |  |  |  |  |  |
| -396 | rs11923810 | 11033501 | Intron2 | T/C | TTAAG**T/C**ACTTA | 1/0 | 0.78/0.22 (0.029-0.411) | 1/0 | 0.87/0.13  (-0.035-0.295) | 1/0 |
|  |  |  |  |  |  |  |  |  |  |  |
| -234 | rs41388950 | 11033663 | Intron2 | T/C | AAGGG**C/T**GGGTG | 0/1 | 0.22/0.78 (0.029-0.411) | 0/1 | 0/1 | 0.44/0.56 (0.197-0.683) |
|  |  |  |  |  |  |  |  |  |  |  |
| -218 | rs11919775 | 11033679 | Intron2 | G/C | AAGAG**G/C**GCTTA | 1/0 | 0.78/0.22 (0.029-0.411) | 0.94/0.06  (-0.056-0.176) | 0.94/0.06  (-0.056-0.176) | 1/0 |
|  |  |  |  |  |  |  |  |  |  |  |
| -17 | rs41362845 | 11033880 | Exon3 (5’UTR) | C/T | CCCTG**C/T**GTCCA | 1/0 | 1/0 | 0.94/0.06  (-0.056-0.176) | 1/0 | 1/0 |
|  |  |  |  |  |  |  |  |  |  |  |
| 949 | rs2928078 | 11034846 | Intron4 | G/A | CAGAA**G/A**CTAGG | 0.43/0.57 (0.171-0.689) | 0.33/0.67 (0.113-0.547) | 0.5/0.5  (0.255-0.745) | 0.56/0.44 (0.197-0.683) | 0.06/0.94  (-0.056-0.176) |
|  |  |  |  |  |  |  |  |  |  |  |
| 949 | rs2928078 | 11034846 | Intron4 | G/A | CAGAA**G/A**CTAGG | 0.47/0.53 (0.368-0.572) | 0.25/0.75  (0.173-0.327) | 0.31/0.69 (0.217-0.403) | 0.62/0.38 (0.292-0.468) | 0.76/0.24 (0.155-0.325) |
|  |  |  |  |  |  |  |  |  |  |  |
| 1063 | rs7643585 | 11034960 | Intron4 | C/T | TGCCT**C/T**GTGCT | 1/0 | 0.83/0.17  (-0.004-0.344) | 1/0 | 1/0 | 0.56/0.44 (0.197-0.683) |
|  |  |  |  |  |  |  |  |  |  |  |
| 1064 | rs41391147 | 11034961 | Intron4 | G/C | GCCTC**G/C**TGCTC | 1/0 | 1/0 | 0.94/0.06 | 1/0 | 1/0 |
|  |  |  |  |  |  |  |  | (-0.056-0.176) |  |  |
| 1076 | rs41444444 | 11034973 | Intron4 | C/T | GTGCC**T/C**GACAG | 0/1 | 0/1 | 0/1 | 0.06/0.94  (-0.056-0.176) | 0/1 |
|  |  |  |  |  |  |  |  |  |  |  |
| 1098 | rs1728803 | 11034995 | Intron4 | A/G | GATAC**A/G**TGGTG | 0.5/0.5  (0.238-0.762) | 0.83/0.17  (-0.004-0.344) | 0.44/0.56 (0.197-0.683) | 0.5/0.5  (0.255-0.745) | 0.81/0.19  (-0.002-0.382) |
|  |  |  |  |  |  |  |  |  |  |  |
| 1114 | rs41381549 | 11035011 | Intron4 | A/C | TGCTC**A/C**CTGAC | 1/0 | 1/0 | 1/0 | 0.94/0.06  (-0.056-0.176) | 1/0 |
|  |  |  |  |  |  |  |  |  |  |  |
| 1532 | rs3817585 | 11035429 | Intron5 | G/C | ACCCA**G/C**AGCCC | 1/0 | 1/0 | 1/0 | 0.87/0.13  (-0.035-0.295) | 0.94/0.06  (-0.056-0.176) |
|  |  |  |  |  |  |  |  |  |  |  |
| 1606 | rs41524646 | 11035503 | Intron5 | G/A | GGTTT**G/A**TCTTT | 1/0 | 0.89/0.11  (-0.035-0.255) | 1/0 | 1/0 | 1/0 |
|  |  |  |  |  |  |  |  |  |  |  |
| 1724 | rs2933307 | 11035621 | Intron5 | G/C | AAGTG**C/G**GTATT | 0.43/0.57 (0.171-0.689) | 0.67/0.33 (0.113-0.547) | 0.50/0.50  (0.255-0.745) | 0.62/0.38 (0.142-0.618) | 0.06/0.94  (-0.056-0.176) |
|  |  |  |  |  |  |  |  |  |  |  |
| 1773 | rs9827626 | 11035670 | Intron5 | C/T | AAATC**C/T**TGACT | 1/0 | 0.78/0.22 (0.029-0.411) | 1/0 | 0.87/0.13  (-0.035-0.295) | 1/0 |
|  |  |  |  |  |  |  |  |  |  |  |
| 1775 | rs2928077 | 11035672 | Intron5 | G/C | ATCCT**G/C**ACTCT | 0.57/0.43 (0.171-0.689) | 0.61/0.39 (0.165-0.615) | 0.5/0.5  (0.255-0.745) | 0.56/0.44 (0.197-0.683) | 0.94/0.06  (-0.056-0.176) |
|  |  |  |  |  |  |  |  |  |  |  |
| 1897 | rs41370144 | 11035794 | Intron5 | C/T | TTAAA**C/T**ATAGA | 1/0 | 1/0 | 1/0 | 1/0 | 0.87/0.13  (-0.035-0.295) |
|  |  |  |  |  |  |  |  |  |  |  |
| 2008 | rs1710888 | 11035905 | Intron5 | C/T | ATTCA**C/T**AGTAG | 1/0 | 1/0 | 1/0 | 0.87/0.13  (-0.035-0.295) | 1/0 |
|  |  |  |  |  |  |  |  |  |  |  |
| 2884 | rs41497953 | 11036781 | Intron5 | C/T | TGCTG**C/T**GAAGG | 1/0 | 0.89/0.11  (-0.035-0.255) | 1/0 | 1/0 | 1/0 |
|  |  |  |  |  |  |  |  |  |  |  |
| 2916 | rs41478447 | 11036813 | Intron5 | C/T | GACCA**T/C**GAGGG | 0/1 | 0.06/0.94  (-0.05-0.17) | 0/1 | 0/1 | 0/1 |
|  |  |  |  |  |  |  |  |  |  |  |
| 3164 | rs3774070 | 11037061 | Intron6 | C/T | GAGCC**C/T**GGCAG | 1/0 | 0.61/0.39 (0.165-0.615) | 0.87/0.13  (-0.035-0.295) | 0.87/0.13  (-0.035-0.295) | 0.94/0.06  (-0.056-0.176) |
|  |  |  |  |  |  |  |  |  |  |  |
| 3164 | rs3774070 | 11037061 | Intron6 | C/T | GAGCC**C/T**GGCAG | 0.94/0.06  (0.011-0.109) | 0.5/0.5  (0.411-0.589) | 0.93/0.07  (0.018-0.122) | 0.89/0.11  (0.054-0.166) | 0.92/0.08  (0.026-0.134) |
|  |  |  |  |  |  |  |  |  |  |  |
| 4647 | rs11712912 | 11038544 | Intron6 | A/G | GCACC**G/A**GCAAA | 0/1 | 0.06/0.94  (-0.05-0.17) | 0/1 | 0.13/0.87  (-0.035-0.295) | 0.06/0.94  (-0.056-0.176) |
|  |  |  |  |  |  |  |  |  |  |  |
| 4766 | rs3856786 | 11038663 | Intron6 | G/A | GCAGA**G/A**GAGTG | 1/0 | 0.94/0.06  (-0.05-0.17) | 1/0 | 0.87/0.13  (-0.035-0.295) | 1/0 |
|  |  |  |  |  |  |  |  |  |  |  |
| 4965 | rs3856787 | 11038862 | Intron6 | G/A | AATGG**G/A**CCTCA | 1/0 | 0.94/0.06  (-0.05-0.17) | 1/0 | 1/0 | 1/0 |
|  |  |  |  |  |  |  |  |  |  |  |
| 5193 | rs6344 | 11039090 | Exon7 | G/T | ATCAC**G/T**CTGGC | 1/0 | 1/0 | 0.94/0.06  (-0.056-0.176) | **a** | **a** |
|  |  |  |  |  |  |  |  |  |  |  |
| 7772 | rs10510403 | 11041669 | Intron7 | A/G | GCATT**A/G**AAGTA | 0.82/0.18  (0.101-0.259) | 0.89/0.11  (0.054-0.166) | 0.86/0.14 (0.070-0.210) | 0.74/0.26 (0.181-0.339) | 0.81/0.19  (0.112-0.268) |
|  |  |  |  |  |  |  |  |  |  |  |
| 7942 | rs41323844 | 11041840 | Intron7 | A/C | TGTGC**A/C**TATGT | 1/0 | 0.89/0.11  (-0.035-0.255) | 1/0 | 0.87/0.13  (-0.035-0.295) | 1/0 |
|  |  |  |  |  |  |  |  |  |  |  |
| 7944 | rs41480248 | 11041842 | Intron7 | A/C | TGCAT**A/C**TGTAA | 1/0 | 0.89/0.11  (-0.035-0.255) | 1/0 | 1/0 | 1/0 |
|  |  |  |  |  |  |  |  |  |  |  |
| 7978 | rs9822125 | 11041875 | Intron7 | A/T | ATGCA**A/T**TACTT | 1/0 | 0.50/0.50 (0.269-0.731) | 1/0 | 0.94/0.06  (-0.056-0.176) | 1/0 |
|  |  |  |  |  |  |  |  |  |  |  |
| 8159 | rs41335049 | 11042056 | Intron7 | C/T | AGATA**C/T**GGATG | 1/0 | 0.83/0.17  (-0.004-0.344) | 1/0 | 1/0 | 1/0 |
|  |  |  |  |  |  |  |  |  |  |  |
| 8229 | rs41537851 | 11042126 | Intron7 | C/T | ACTTT**C/T**CTCCC | 1/0 | 0.94/0.06  (-0.05-0.17) | 1/0 | 1/0 | 1/0 |
|  |  |  |  |  |  |  |  |  |  |  |
| 8302 | rs33948309 | 11042199 | Exon8 | G/A | GTGAC**G/A**CTGCC | 1/0 | 0.89/0.11  (-0.035-0.255) | 1/0 | 1/0 | 1/0 |
|  |  |  |  |  |  |  |  |  |  |  |
| 8443 | rs17532365 | 11042340 | Intron8 | G/A | TTTCT**G/A**ACCCTC | 1/0 | 1/0 | 0.87/0.13  (-0.035-0.295) | 1/0 | 1/0 |
|  |  |  |  |  |  |  |  |  |  |  |
| 8830 | rs41426745 | 11042727 | Intron9 | G/C | TTCCT**G/C**TTGTC | 1/0 | 0.83/0.17  (-0.004-0.344) | 1/0 | 1/0 | 1/0 |
|  |  |  |  |  |  |  |  |  |  |  |
| 9029 | rs35972647 | 11042926 | Exon10 | C/T | GACTC**C/T**ATCAT | 1/0 | 0.83/0.17  (-0.004-0.344) | 1/0 | 1/0 | 1/0 |
|  |  |  |  |  |  |  |  |  |  |  |
| 11967 | rs6770472 | 11045863 | Intron11 | G/T | TTGGG**G/T**CTGGG | 1/0 | 0.94/0.06  (-0.05-0.17) | 1/0 | 1/0 | 1/0 |
|  |  |  |  |  |  |  |  |  |  |  |
| 11973 | rs36034065 | 11045869 | Intron11 | G/A | CTGGG**G/A**CTGCT | 1/0 | 0.89/0.11  (-0.035-0.255) | 1/0 | 1/0 | 1/0 |
|  |  |  |  |  |  |  |  |  |  |  |
| 13269 | rs11925331 | 11047166 | Intron12 | C/T | CAGCA**C/T**AGAGA | 0.96/0.04  (0-0.080) | 0.66/0.34 (0.255-0.425) | 0.99/0.01  (-0.010-0.030) | 0.68/0.32 (0.236-0.404) | 0.65/0.35 (0.255-0.445) |
|  |  |  |  |  |  |  |  |  |  |  |
| 14351 | rs2272403 | 11048248 | Intron13 | G/A | ACACA**G/A**TCTAG | 0.93/0.07  (-0.064-0.204) | 0.44/0.56 (0.211-0.669) | 0.81/0.19  (-0.002-0.382) | 0.5/0.5  (0.255-0.745) | 0.69/0.31 (0.083-0.537) |
|  |  |  |  |  |  |  |  |  |  |  |
| 14351 | rs2272403 | 11048248 | Intron13 | G/A | ACACA**G/A**TCTAG | 0.88/0.12  (0.054-0.186) | 0.38/0.62 (0.293-0.467) | 0.89/0.11  (0.047-0.173) | 0.62/0.38 (0.292-0.468) | 0.64/0.36 (0.264-0.456) |
|  |  |  |  |  |  |  |  |  |  |  |
| 16009 | rs41436650 | 11049906 | Intron13 | A/G | CAGGC**A/G**TGGGC | 1/0 | 0.89/0.11  (-0.035-0.255) | 0.94/0.06  (-0.056-0.176) | 1/0 | 0.87/0.13  (-0.035-0.295) |
|  |  |  |  |  |  |  |  |  |  |  |
| 16009 | rs41436650 | 11049906 | Intron13 | A/G | CAGGC**A/G**TGGGC | 0.97/0.03  (-0.005-0.065) | 0.89/0.11  (0.054-0.166) | 0.98/0.02  (-0.008-0.048) | 0.94/0.06  (0.017-0.103) | 0.80/0.20 (0.120-0.280) |
|  |  |  |  |  |  |  |  |  |  |  |
| 16116 | rs2675163 | 11050013 | Intron13 | C/T | GATGT**C/T**GAGTG | 0.29/0.71 (0.052-0.528) | 0.11/0.89  (-0.035-0.255) | 0.19/0.81  (-0.002-0.382) | 0.19/0.81  (-0.002-0.382) | 0.69/0.31 (0.083-0.537) |
|  |  |  |  |  |  |  |  |  |  |  |
| 16116 | rs2675163 | 11050013 | Intron13 | C/T | GATGT**C/T**GAGTG | 0.25/0.75 (0.162-0.338) | 0.14/0.86  (0.078-0.202) | 0.20/0.80 (0.119-0.281) | 0.27/0.73 (0.190-0.350) | 0.51/0.49 (0.390-0.590) |
|  |  |  |  |  |  |  |  |  |  |  |
| 16605 | rs2246543 | 11050502 | Intron14 | T/C | GAAAC**T/C**TCTAG | 0.64/0.36 (0.109-0.611) | 0.94/0.06  (-0.05-0.17) | 0.81/0.19  (-0.002-0.382) | 0.25/0.75 (0.038-0.462) | 0/1 |
|  |  |  |  |  |  |  |  |  |  |  |
| 16605 | rs2246543 | 11050502 | Intron14 | T/C | GAAAC**T/C**TCTAG | 0.53/0.47 (0.368-0.572) | 0.86/0.14  (0.078-0.202) | 0.43/0.57 (0.330-0.530) | 0.25/0.75 (0.172-0.328) | 0.17/0.83  (0.095-0.245) |
|  |  |  |  |  |  |  |  |  |  |  |
| 17502 | rs35957531 | 11051399 | Intron15 | C/G | CCCCA**C/G**CCTTC | 0.93/0.07  (-0.064-0.204) | 1/0 | 0.75/0.25 (0.038-0.462) | 1/0 | 1/0 |
|  |  |  |  |  |  |  |  |  |  |  |
| 17885 | rs41517144 | 11051810 | Intron15 | A/C | ATGAC**A/C**AGAGA | 1/0 | 0.78/0.22 (0.029-0.411) | 1/0 | 1/0 | 1/0 |
|  |  |  |  |  |  |  |  |  |  |  |
| 17913 | rs2697138 | 11051906 | Intron15 | G/T | ATGGG**G/T**TGTCA | 0.93/0.07  (-0.064-0.204) | 1/0 | 0.75/0.25 (0.038-0.462) | 0.81/0.19  (-0.002-0.382) | 0.62/0.38 (0.142-0.618) |
|  |  |  |  |  |  |  |  |  |  |  |
| 19475 | rs2697134 | 11053372 | Intron15 | G/A | GTGGG**G/A**CAGTG | 0.93/0.07  (-0.064-0.204) | 1/0 | 0.75/0.25 (0.038-0.462) | 0.69/0.31 (0.083-0.537) | 0.06/0.94  (-0.056-0.176) |
|  |  |  |  |  |  |  |  |  |  |  |
| 20172 | rs2675165 | 11054069 | Exon16 (3’UTR) | C/T | CCCTC**C/T**GAACG | 0.93/0.07  (-0.064-0.204) | 1/0 | 0.75/0.25 (0.038-0.462) | 0.81/0.19  (-0.002-0.382) | 0.56/0.44 |
|  |  |  |  |  |  |  |  |  |  | (0.197-0.683) |
| 20172 | rs2675165 | 11054069 | Exon16 (3’UTR) | C/T | CCCTC**C/T**GAACG | 0.83/0.17  (0.093-0.247) | 0.97/0.03  (-0.001-0.061) | 0.84/0.16 (0.086-0.234) | 0.81/0.19  (0.119-0.261) | 0.80/0.20 (0.120-0.280) |
|  |  |  |  |  |  |  |  |  |  |  |
| 20177 | rs41510044 | 11054074 | Exon16 (3’UTR) | A/G | CGAAC**G/A**CTGCT | 0.5/0.5  (0.238-0.762) | 0.39/0.61 (0.165-0.615) | 0.13/0.87  (-0.035-0.295) | 0/1 | 0/1 |
|  |  |  |  |  |  |  |  |  |  |  |
| 20358 | rs2675166 | 11054255 | Exon16 (3’UTR) | C/T | TATTC**C/T**CAGGG | 0.93/0.07  (-0.064-0.204) | 1/0 | 0.75/0.25 (0.038-0.462) | 0.81/0.19  (-0.002-0.382) | 0.56/0.44 (0.197-0.683) |
|  |  |  |  |  |  |  |  |  |  |  |
| 20622 | rs2944367 | 11054519 | Exon16 (3’UTR) | A/G | GCCCA**A/G**TTTCA | 0.71/0.29 (0.052-0.528) | 0.61/0.39 (0.165-0.615) | 0.75/0.25 (0.038-0.462) | 0.75/0.25 (0.038-0.462) | 0.62/0.38 (0.142-0.618) |
|  |  |  |  |  |  |  |  |  |  |  |
| 20622 | rs2944367 | 11054519 | Exon16 (3’UTR) | A/G | GCCCA**A/G**TTTCA | 0.74/0.26  (0.170-0.350) | 0.84/0.16  (0.094-0.226) | 0.79/0.21 (0.128-0.292) | 0.84/0.16  (0.094-0.226) | 0.81/0.19  (0.112-0.268) |
|  |  |  |  |  |  |  |  |  |  |  |
| 21271 | rs1062246 | 11055168 | Exon16 (3’UTR) | G/A | CTCAC**A/G**TGTGG | 0.36/0.64 (0.109-0.611) | 0.56/0.44 (0.211-0.669) | 0.5/0.5  (0.255-0.745) | 0.44/0.56 (0.197-0.683) | 0.62/0.38 (0.142-0.618) |
|  |  |  |  |  |  |  |  |  |  |  |
| 21726 | rs41276505 | 11055624 | Exon16 (3’UTR) | C/T | TTGCC**C/T**GGGGG | 0.93/0.07  (-0.064-0.204) | 1/0 | 1/0 | 1/0 | 1/0 |
|  |  |  |  |  |  |  |  |  |  |  |

SNPs genotyped in the population samples are also provided. A total of 61 SNPs and two length polymorphisms were discovered. Name refers to the position of the polymorphism in relation to the ATG of the *SLC6A1* gene. Column “rs” gives reference SNP number is it was available. Location refers to the location of the polymorphism in the gene. In columns “EA,” “AA,” “Finn,” “Thai,” “Hmong,” allele frequencies of the SNP, estimated based on sequencing of 14-18 chromosomes in each population, is given. “a” no sequence data available for this segment. “b” = frequency was calculated based on the frequency of –GG homozygote and assuming Hardy-Weinberg Equilibrium. “( )”= 95% Confidence Interval for of the minor allele frequency. Highlighted rows = Frequency data obtained by genotyping the SNP in larger samples of Thai (n=59), Hmong (n=48), European-American (n=46), African-American (n=60) and Finnish subjects (n=47). For example, SNP rs34189945 was detected both by sequencing and genotyping and thus listed twice in this table.
